# Independent Neural Activity Patterns for Sensory- and Confidence-Based Information Maintenance during Category-Selective Visual Processing

**DOI:** 10.1523/ENEURO.0268-18.2018

**Published:** 2019-02-22

**Authors:** Matthew D. Weaver, Johannes J. Fahrenfort, Artem Belopolsky, Simon van Gaal

**Affiliations:** 1 Department of Psychology, University of Amsterdam, Amsterdam 1001 NK, The Netherlands; 2 Amsterdam Brain and Cognition (ABC), University of Amsterdam, Amsterdam 1001 NK, The Netherlands; 3 Experimental and Applied Psychology, Vrije Universiteit Amsterdam, Amsterdam 1081 BT, The Netherlands

**Keywords:** consciousness, decision confidence, EEG decoding

## Abstract

Several influential theories of consciousness attempt to explain how, when and where conscious perception arises in the brain. The extent of conscious perception of a stimulus is often probed by asking subjects to provide confidence estimations in their choices in challenging perceptual decision-making tasks. Here, we aimed to dissociate neural patterns of “cognitive” and “sensory” information maintenance by linking category selective visual processes to decision confidence using multivariate decoding techniques on human EEG data. Participants discriminated at-threshold masked face versus house stimuli and reported confidence in their discrimination performance. Three distinct types of category-selective neural activity patterns were observed, dissociable by their timing, scalp topography, relationship with decision confidence, and generalization profile. An early (∼150–200 ms) decoding profile was unrelated to confidence and quickly followed by two distinct decodable patterns of late neural activity (350–500 ms). One pattern was on*-*diagonal, global and highly related to decision confidence, likely indicating cognitive maintenance of consciously reportable stimulus representations. The other pattern however was off-diagonal, restricted to posterior electrode sites (local), and independent of decision confidence, and therefore may reflect sensory maintenance of category-specific information, possibly operating via recurrent processes within visual cortices. These results highlight that two functionally independent neural processes are operating in parallel, only one of which is related to decision confidence and conscious access.

## Significance Statement

The aim of the present study was to dissociate neural patterns of “cognitive” (confidence dependent) and “sensory” (confidence independent) category-selective information maintenance using multivariate decoding techniques on human EEG data. We found evidence of two functionally independent feedback-related neural processes operating in parallel. One pattern was global and related to decision confidence, likely indicating cognitive maintenance of consciously reportable stimulus representations. The other pattern was restricted to posterior electrode sites and unrelated to decision confidence, indicating sensory maintenance of category-specific information within visual cortices.

## Introduction

The brain hosts a massive parallel processing pipeline for the extraction of sensory input (Wolfe, 1994; Corbetta and Shulman, 1998; Coull and Nobre, 1998; Itti and Koch, 1999; Vagharchakian et al., 2012; Marti et al., 2015). Part of this processing machinery unfolds independently of subjective awareness, whereas other processes may not. To illustrate, fully masked (i.e., imperceptible) images still activate many (sub)cortical modules, without enabling conscious access (Dehaene and Changeux, 2011; Kunde et al., 2012; van Gaal and Lamme, 2012; Boly et al., 2013). Therefore, it is clear that mere activation of a specific brain region does not support conscious perception. Although considerable controversy exists, a broad scientific consensus contends that feedback processes, either at a local or more global scale, support the brain’s capacity to maintain and integrate information over space and time and are crucial for conscious perception and report (Dehaene and Naccache, 2001; Lamme, 2006; Tononi and Koch, 2008).

Here, we aim to dissociate the neural patterns of “sensory” and “cognitive” category-selective information maintenance by linking perceptual processes to decision confidence (i.e., metacognition), a proxy for stimulus reportability (Galvin et al., 2003; Kepecs et al., 2008; Kiani and Shadlen, 2009; Resulaj et al., 2009). Previous studies have revealed that confidence estimations, or metacognition in general, are mediated by (anterior parts of) the prefrontal cortex (Rounis et al., 2010; Fleming and Dolan, 2012), and that damage to these regions impairs metacognitive insight (Fleming et al., 2014). By determining how closely an observer’s confidence differentiates correct from incorrect perceptual decisions, confidence reports can be used to calculate metacognitive sensitivity (meta-*d’*), which reflects the extent to which the observer is able to reflect on their own cognitive processes (Maniscalco and Lau, 2012; Fleming and Lau, 2014).

**Figure 1.**
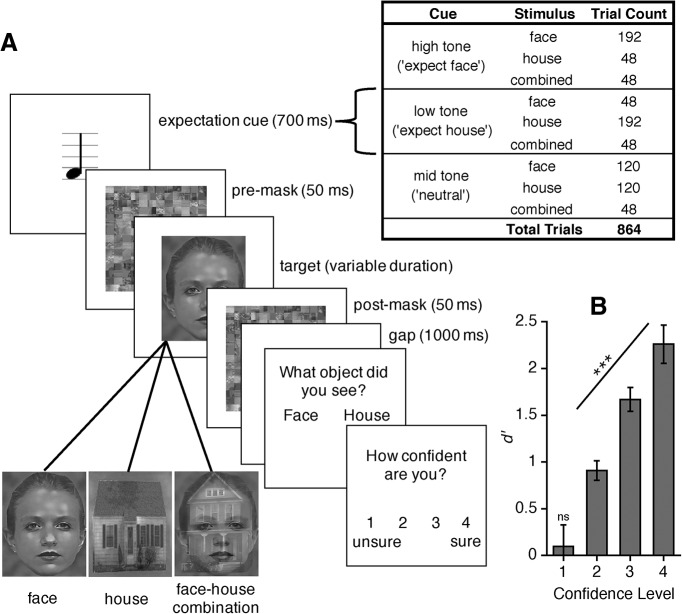
***A***, Trial sequence. Participants discriminated masked face and house stimuli and reported their confidence in this decision. Participants were naïve to the presentation of an additional face-house combination category on some trials. Preceding tones induced different expectations about the likelihood of presented stimulus category on a given trial, according to explicit tone-stimulus contingencies. ***B***, Behavioral results. *d’* for discrimination responses as a function of reported confidence; ***p < 0.001, ns: not significant.

Here, we presented masked faces or masked houses and asked participants on every trial which stimulus category they perceived and how confident they were in this decision (Fig. 1*A*). First-order task performance was individually titrated at 75% correct. A multivariate pattern analysis (MVPA) classifier was trained and tested on EEG activity to discriminate face versus house stimuli, separately for high-confidence and low-confidence decisions. Crucially, each classifier was trained on EEG activity at one time sample and tested on activity at all other time samples, allowing us to explore how category-selective neural representations generalize across time. We aimed to isolate those category-selective neural signals that depend on decision confidence, reflecting cognitive maintenance of information, from those neural signals that may be independent of confidence, reflecting pure sensory maintenance of information. We were also interested in how expectation may modulate and bias category-selective neural processing and perceptual interpretation. Accordingly, we included bi-interpretable stimuli, which were constructed by overlapping a face and a house stimulus. Because each trial was preceded by a tone predicting the likelihood that a face or a house stimulus would appear we could explore how expectations might bias perceptual interpretation and neural representations when confronted with ambiguous visual input (Kok et al., 2012; Pinto et al., 2015; Aru et al., 2016; Meijs et al., 2018).

## Materials and Methods

### Participants

Twenty-five volunteers participated in exchange for cash or course credit. All participants gave informed consent and were naïve to the specific hypotheses. Participants had either normal or corrected-to-normal vision (17 females, 23 right-handed, mean age of 22.92 years). All procedures were approved by the ethics committee.

### Experimental setting

The experiment was programmed and executed using Psychophysics Toolbox (version 3.0.14; Brainard, 1997) within the MATLAB environment (R2010, MathWorks, Inc.). Stimuli were presented on an Asus VG236H LCD monitor (23” diagonal, 1920 × 1080-pixel resolution; 100-Hz refresh rate) at a viewing distance of 63 cm.

### Experimental design, procedure, and stimuli

Figure 1*A* shows an overview of the trial procedure. Each trial began with a central fixation point. After 150–650 ms, a cue tone sounded for 200 ms. The tone pitch indicated the likelihood that the upcoming target stimulus would belong to a particular category (face or house). The target image was presented 750 ms after cue tone onset for a variable duration (see titration procedure below). Target stimuli were immediately preceded and followed by scrambled masks presented for 50 ms. A response screen was presented 1000 ms after the second mask offset, instructing participants to choose whether the presented image was a face or a house using a left-handed (“z”) or right-handed (“m”) keyboard response. Stimulus-response mappings were indicated by whether “face” and “house” labels were shown in the left or right hemifield, and were randomized on each trial to prevent motor response preparation before the response screen. Participants then made a second response to indicate how confident they were in the accuracy of their discrimination response: a “1” (“unsure”), “2,” “3,” or “4” (“sure”) keyboard press. Trials ended if no response was recorded within 5000 ms of either response screen. Participants completed 864 experimental trials, separated into 12 blocks. Experimental conditions were counterbalanced within blocks.

All stimuli subtended 16 × 20° visual angle, were greyscale, and were centrally presented on a black background. Target stimuli consisted of 180 unique house and 180 unique face images (90 male, 90 female, each presented twice during the experiment) and with 144 bi-interpretable face-house images (each presented once). Bi-interpretable images were created by randomly matching each unique face image to a house image, making each stimulus 50% transparent and superimposing them. Five sets of 180 bi-interpretable images were constructed. One set was selected per participant (counterbalanced across participants), from which 144 images were randomly selected for presentation. Bi-interpretable stimuli were not presented during practice and participants were not informed that such stimuli would be presented. Because bi-interpretable stimuli contained both face and house features, they were not included in discrimination performance measures. Masks were randomly selected from 900 images comprised of scrambled face and house images (parsed into 12 × 15 tiles and randomly shuffled) that had been made transparent and superimposed. The SHINE toolbox (Williams et al., 2009) was used to equate all face, house, and bi-interpretable stimuli for spatial frequency (equating rotational average of the Fourier amplitude spectrum) and then for luminance (equating luminance histograms) over 20 iterations. House stimuli were taken from Egner et al. (2010), and face stimuli were compiled from the Cohn–Kanade Facial Expression Database (Kanade and Cohn, 2000) and from Endl et al. (1998).

We titrated target image presentation duration to achieve 75% discrimination accuracy. Duration was initially titrated trial-by-trial during 60 practice trials using a weighted 3-up-1-down staircase procedure (Kaernbach, 1991) with a step size of 10 ms and an initial duration of 80 ms. Target duration for the first experimental block was derived from this practice, resulting in initial target durations ranging from 10–30 ms across participants. Target duration was then held consistent within experimental blocks. If discrimination accuracy was above 80% for a given block, target duration decreased by 10 ms for the subsequent block; if accuracy dropped below 70%, duration increased by 10 ms.

High-pitch (1800 Hz) and low-pitch (400 Hz) cue tones validly predicted the appearance of their respective stimulus categories with 80% accuracy, creating expectation conditions based on whether the target stimulus could be expected (i.e., valid cue) or unexpected (i.e., invalid cue) on any given trial. A mid-pitch cue tone (1000 Hz) always indicated an equal likelihood of either stimulus category being presented (neutral cue condition). Each cue tone was equally likely, across both single category and bi-interpretable stimuli trials, and tone-stimuli category likelihood mappings were counterbalanced across participants. Participants were explicitly informed about tone-stimulus contingencies but were instructed to make their discrimination response based on their perception of the actual image presented.

### EEG recording and preprocessing

EEG was recorded using the 64-channel BioSemi ActiveTwo system (BioSemi) and digitized at a 512-Hz sample rate. Sixty-four scalp electrodes arranged according to the 10-20 system (Jasper, 1958) were measured, along with two reference electrodes on the earlobes. EEG preprocessing and analyses were conducted using custom scripts, the EEGLAB toolbox (v13.1.1; Delorme and Makeig, 2004), and the Amsterdam Decoding and Modeling toolbox (ADAM; Fahrenfort et al., 2018). Data were re-referenced offline to the linked earlobes, high-pass filtered at 0.1 Hz (cutoff frequency: –6 dB at 0.05 Hz), and epoched from –500 to 2500 ms surrounding cue onset. EEG activity was baseline corrected using the 200-ms interval preceding cue onset. Trials containing muscle artefacts within the 250-ms interval following target onset were removed using an adapted version of the ft_artifact_zvalue muscle artifact detection function from the FieldTrip toolbox (Oostenveld et al., 2011). This function applies a frequency filter between 110 and 140 Hz and assigns a z-value to each time sample to determine the degree to which power values in that frequency range deviate from normality. Muscle artefacts were identified as *z* score outliers >3 SDs from absolute value of the minimum negative *z* value. This resulted in removal of 2.61% of trials. Data were then downsampled to 128 Hz.

### Statistical analyses

#### Behavior

Additional trials were discarded if participants made an anticipatory discrimination response (<200 ms; 0.25%) or offered no response (0.51%). We used D-prime (*d’*; Type-I sensitivity) as a bias-free measure of perceptual sensitivity to the stimulus and metacognitive (meta-*d’*) efficiency as a measure of a participant’s metacognitive capacity given a particular level of task performance (for detailed description, see Fleming and Lau, 2014). Meta-*d’* (Type-II sensitivity) measures the degree to which participants are consciously aware of the accuracy of their discrimination judgments, as indicated by their confidence responses. Meta-*d’* is constrained by a participant’s *d’* performance, such that a metacognitively ideal observer will theoretically have a meta-*d’* equal to their *d’*, while a suboptimal observer will have a meta-*d’* less than *d’*. Meta-*d’* efficiency was calculated as meta-*d’* minus *d’*, thus higher values (i.e., smaller negative values approaching zero) indicate more metacognitively efficient performance.

### EEG MVPA/decoding

A decoding classification algorithm using a 10-fold cross validation scheme was applied to each participant’s data. First, we randomized the order of trials and split the dataset into 10 equally sized subsets. Face and house stimulus classes were balanced in the training set by duplicating underrepresented stimulus class instances at random to match the number of instances of the largest stimulus class. We then trained a linear discriminant classifier to discriminate between face and house stimulus classes using 90% of the data, then tested it on the remaining 10% of the data to ensure independence of training and testing sets. This process was repeated 10 times to test all the data once. Features for classification consisted of EEG amplitudes of individual electrodes. Classification accuracy was calculated for each participant by first averaging the proportion of correct class assignments for each stimulus category, then averaging across stimulus categories, and finally averaging across the 10 folds. This cross-validation procedure was repeated so that the algorithm was trained on activity at each time sample (e.g., *t_1_*) and then tested on activity at every time sample (*t_1_*, *t_2_*, *t_3_*, …), creating a temporal generalization matrix of classification accuracies at each possible combination of training and testing time samples (King and Dehaene, 2014). The *y*-axis on the matrix presents the time when the classifier is trained and the *x*-axis presents the time when the classifier is tested.

**Figure 2.**
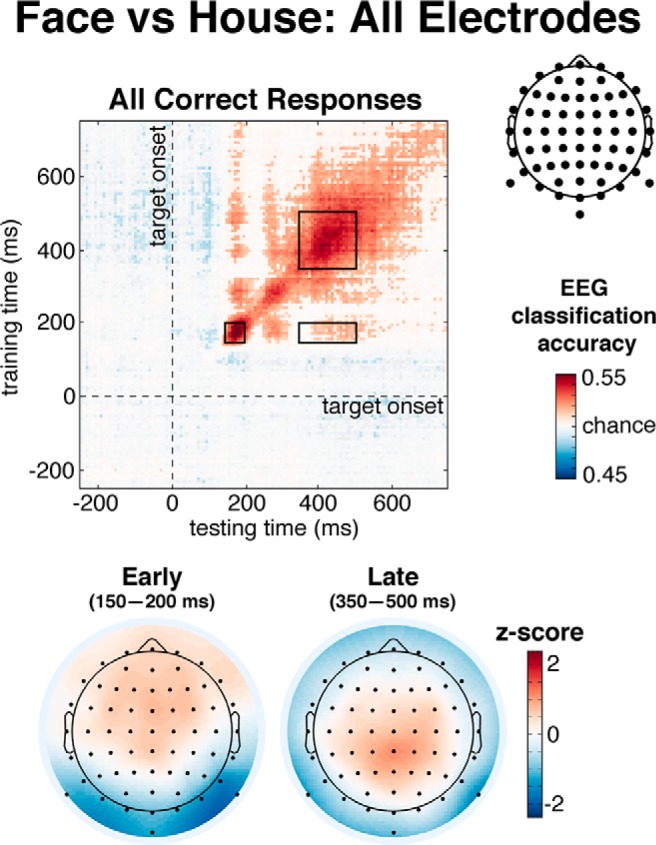
Temporal generalization matrix of classification accuracies for face versus house discrimination across all electrodes. The *y*-axis depicts when classifier was trained, and *x*-axis depicts when classifier was tested, relative to target stimulus onset. Values not significantly different from chance are masked. ROIs are denoted by inset black boxes. Below, correlation/class separability maps for each ROI, revealing underlying neural sources. Note, because such maps are based on training data, the off-diagonal rectangle ROI map would be comparable to that of the early ROI.

To keep the data and analytical strategy separate (Kriegeskorte et al., 2009), we restricted analyses to two non-overlapping electrode sets based on the international 10-20 EEG placement system nomenclature (Jasper, 1958). The occipital-parietal set included occipital, occipito-parietal and parietal electrodes, chosen to capture early visual and parietal “N170-like” activity (Iz, Oz, O1, O_2_, POz, PO3, PO4, PO7, PO8, Pz, P1, P2, P3, P4, P5, P6, P7, P8, P9, P10). The frontal-central set included central, fronto-central, and frontal electrodes, chosen to capture late frontal category-selective representations related to confidence (Cz, C1, C2, C3, C4, FCz, FC1, FC2, FC3, FC4, Fz, F1, F2, F3, F4; Del Cul et al., 2007; Rounis et al., 2010; Fleming and Dolan, 2012; Fleming et al., 2014; Marti and Dehaene, 2017). To check whether the observed effects were due to our electrode selection method, we also performed a control analysis using a more data-driven approach. All significant correlations, main effects, and the interaction between confidence and generalization type (on-diagonal vs off-diagonal) observed for the Frontal-Central set also held when selecting the data-driven set of electrodes that best discriminated between face versus house stimuli (i.e., capturing the more posterior P3-like late activity observable in Fig. 2, bottom-right; POz, Pz, P1, P2, P3, P4, P5, CPz, CP1, CP2, CP3, CP4, Cz, C1, C2, C3, C4, FCz, FC1, FC2, FC3, FC4).

**Figure 3.**
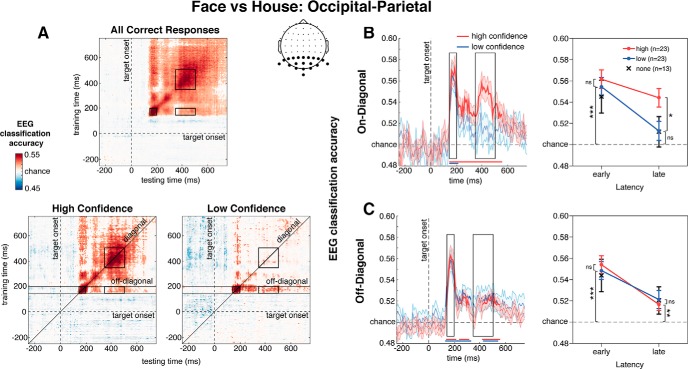
***A***, Temporal generalization matrices of classification accuracies for face versus house discrimination for occipital-parietal electrodes. On-diagonal values indicate time-specific values; off-diagonal values reflect cross-temporal generalization. Data restricted to (***B***) on-diagonal and (***C***) off-diagonal planes of classification accuracy, with classification performance indicated on the *y*-axis. In left panels, bold colored lines indicate when above-chance classification accuracy was observed (*p* < 0.05, cluster-based permutation test). Black boxes designate the same ROIs as for the matrices. Right panels show mean decoding accuracy values derived from within each ROI and used for statistical analyses. Black crosses denote decoding accuracy values when participants were least confident (1, cf. low confidence: 1 and 2). Note, only 13 participants had sufficient data to complete classification procedure for “no confidence” (label: “none”) condition and so it is included here only as a reference; *p < 0.05, **p < 0.01, ***p < 0.001, ns: not significant.

**Figure 4.**
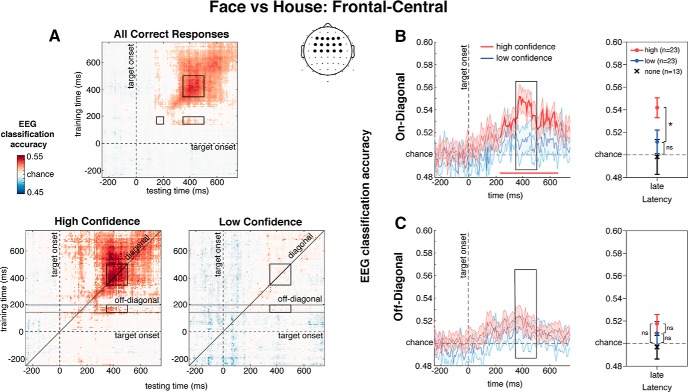
***A***, Temporal generalization matrices of classification accuracies for face versus house discrimination for frontal-central electrodes. Data restricted to (***B***) on-diagonal and (***C***) off-diagonal planes of classification accuracy. Right panels show mean decoding accuracy values derived from within each ROI; *p < 0.05.

To uncover category-selective neural representations with high signal-to-noise ratio (SNR), primary analyses were based only on correct discrimination response trials. However, we also report analyses using all (correct and incorrect) trials to demonstrate that our selection method did not affect our conclusions. First, classification was conducted using each electrode set on all correct trials, followed by separate classifications on correct trials with low confidence (1 or 2 response; to compute classifier accuracy of discriminating between faces and houses when making responses with low confidence responses) and with high confidence (3 or 4 response; to compute classifier accuracy of discriminating between faces and houses when making responses with high confidence). For these latter analyses, we always used the same classifier, training each fold using 90% of all correct responses regardless of whether these were low or high confident, but testing on an independent 10% of either only the relevant low- or the high-confidence subset of the data. Thus, while stimulus class (face vs house) was balanced in the training set, confidence (high vs low) was not. Two participants were excluded from these confidence-specific MVPA analyses for having a minimum number of observations per condition of <10. We also tested the classifier on the lowest indicated confidence response (1), but only 13/25 participants had sufficient trials per condition. Consequently, no formal analyses were conducted, although the classifier accuracy values are included in Figures 3*B*, 4*B* as a reference. As a control analyses, we also tested the classifier on the two highest confidence responses separately (3 and 4).

To determine whether a classifier could discriminate neural activity elicited by a bi-interpretable stimulus solely based on the expectation generated by the cue, we trained a classifier on the entire dataset (cf. 90%) where a correctly identified single face or house stimulus was presented (as above). We then tested the accuracy of the classifier in discriminating between bi-interpretable stimuli preceded by a face versus a house cue (i.e., “expect face” vs “expect house”). Finally, to determine whether expectation itself was decodable, we trained (90%) and tested (10%, 10 folds) on expected versus unexpected correct trials, that is, whether the cue validly or invalidly predicted the stimulus category. These latter classification procedures trained and tested the algorithm on the same time sample only (i.e., on-diagonal decoding, see description below).

Topographic maps were computed by multiplying the corresponding data correlation matrix with the classifier weights to create a correlation/class separability map. Such maps reflect the part of the signal that is strongly correlated with the discrimination of the target variables (faces and houses), while discarding high amplitude artefacts such as blinks even if these have a small (non-zero) correlation with the target variables. For a more detailed description, see Haufe et al. (2014). Accordingly, nonzero activity on these maps reflects electrodes where the face versus house discrimination signal was both strong and highly correlated with the task, thereby minimizing potential influence of Type I and II artifacts and allowing interpretation of neural sources. Maps were then normalized across electrodes for each participant so that the mean signal across electrodes was always zero (similar to taking the average reference when re-referencing). Such a spatial normalizing procedure much improves the ability to average scalp maps across subjects without uneven contribution to signal strength from different subjects, but does make it harder to interpret the polarity of given electrodes per se. Next, to select a data-driven set of electrodes for which to replicate our findings, we calculated significant nonzero activity by conducting *t* tests for each electrode against zero and correcting using cluster-based permutation tests (1000 iterations, 0.05 threshold). The sum of *t* values in an observed cluster of contiguously significant electrodes was compared to the sum of contiguously significant electrodes under random permutation. The same cluster-based permutation method was applied, using time samples instead of electrodes, when highlighting significant time intervals of above-chance decoding performance for the on-diagonal and off-diagonal plots in Figures 3*B*, 4*B* , left panels.

Temporal dependency dynamics of decoding were investigated by examining classification accuracies along the diagonal plane (train on *t_1_*/test on *t_1_*, train on *t_2_*/test on *t_2_*, …) or an off-diagonal/horizontal plane (e.g., train on *t_1_*/test on *t_1_*, *t_2_*, *t_3_*, …) of the temporal generalization matrix. Decoding accuracy along the time-specific diagonal plane reveals when, and for how long, the same information is decodable over time, but cannot distinguish between whether decoding over time is supported by the same neural processes or rather a dynamic chain of distinct processes. However, off-diagonal decoding accuracy across time samples identifies whether patterns of decodable activity generalize to other time samples along a horizontal plane, thus revealing the degree to which underlying neural representations that support ongoing decoding are qualitatively similar or distinct (King and Dehaene, 2014; Stokes, 2015; King et al., 2016). By training the classifier at the time when we expect to observe the earliest category-selective processing (150–200 ms) and testing it on successive time samples, we can use off-diagonal decoding to observe whether initial stimulus-specific processing persists/re-activates over time and if so, how it interacts with reported confidence. Together, we can use these complementary dimensions to provide a comprehensive profile of the presence and dynamic development of category-selective representations during high-confidence and low-confidence perceptual decisions.

We examined decoding accuracy over two preselected latency windows. As for the off-diagonal training time interval, an early latency window (150–200 ms) was based on when we expected initial category-selective stimulus processing to peak (Bentin et al., 1996; Rossion and Jacques, 2011; Carlson et al., 2013; Kaiser et al., 2016; Marti and Dehaene, 2017). A later latency window (350–500 ms) was expected to capture processes associated with more global, stable and accessible representations of semantic category that participants would use to select a response (Kaiser et al., 2016; Marti and Dehaene, 2017). Mean classification performance was taken across these windows. Based on prior expectations (derived from Del Cul et al., 2007; Marti and Dehaene, 2017), only the later latency window was examined for frontal-central electrodes as initial stimulus-specific processes were predicted to only occur over occipital-parietal electrodes.

## Results

To anticipate our findings, confidence reports were found to reliably track perceptual discrimination and we observed the existence of three distinct category-selective representations of a stimulus that were differentially related to decision confidence. However, there was little evidence, behaviorally or electrophysiologically, for any impact of expectation on the processing of bi-interpretable stimuli. Accordingly, we detail the confidence findings first and summarize the predominantly null findings of expectation at the end of the results section.

### Confidence results

#### Behavior

Overall, participants correctly discriminated 75.25% of the single-category images (SD = 2.57%). A one-way repeated measures ANOVA (rANOVA) was conducted on perceptual sensitivity, signal detection theoretic *d’,* to the masked image category using reported confidence level as the within-participants factor. We observed that *d’* increased monotonically as a function of the reported confidence level (Fig. 1*B*). When participants indicated a higher level of confidence, they were better able to perceptually discriminate the image (*F*_(1.63,39.09)_ = 37.51, MSE = 1.08, *p* < 0.001). *d’* was not significantly above zero at the lowest confidence level (M = 0.10, SD = 1.13; *t*_(24)_ = 0.41, *p* = 0.684). These findings reveal that participants were able to accurately introspect their discrimination performance.

### Decoding category-selective electrophysiological responses

Figure 2 shows the temporal generalization matrix of classification accuracy when trained and tested on correctly identified face versus house stimuli, generalized across training and testing time samples, and time-locked to stimulus onset. These results thus reflect only stimulus category decodability and not confidence. First, as expected, we see an early focused peak of decoding accuracy (small square) ∼150–200 ms after stimulus onset, the latency range of the N170, and most prominently over occipital-parietal electrodes (Carlson et al., 2013; Kaiser et al., 2016; Marti and Dehaene, 2017). Second, later, a sustained and stable decoding pattern (large square-shaped pattern) was observed on the diagonal of the generalization matrix with a central-posterior topography, similar to the P3 ERP topography (for review, see Polich, 2011). Third, a modest level of accurate decoding performance is also observed off-diagonal during this same late latency (rectangle-shaped pattern), and indicates that the pattern of activity observed here around 350–500 ms is similar to the activity pattern trained on the early latency window. This off*-*diagonal decoding pattern, which has also previously been observed using MEG (Marti and Dehaene, 2017), suggests sensory maintenance of category-specific information. In follow-up analyses we split these temporal generalization analyses according to scalp topography (posterior vs anterior) and decision confidence (high vs low).

### Occipital-parietal electrodes

We first aimed to statistically determine how category-selective decoding of perceptual brain responses was related to confidence. To do so, we performed an rANOVA with the factors confidence (high vs low), the latency of EEG decoding (early vs late) and temporal generalization type (on-diagonal vs off-diagonal) on decoding accuracy for the occipital-parietal electrode set (Fig. 3, inset). We observed a significant three-way interaction (*F*_(1,22)_ = 16.24, MSE < 0.01, *p* < 0.001), a confidence by generalization interaction (*F*_(1,22)_ = 16.86, MSE < 0.01, *p* < 0.001), as well as significant main effects of generalization type and decoding latency (*F*s > 7.92; all other *F*s < 1.14). We carefully unpack these results in follow-up analyses.

In Figure 3*A*, we show the generalization matrices separately for high confidence (bottom left panel) and low confidence decisions (bottom right panel). The temporal profiles of these matrices reveal a combination of early decoding (150–200 ms), on-diagonal square-shaped decoding (350–500 ms), and off-diagonal rectangle-shaped decoding (350–500 ms). Visual inspection of these temporal generalization matrices shows a clear drop in decoding performance for low confidence decisions, compared to high confidence decisions, related to the on-diagonal square-shaped late decoding performance. In contrast, early on-diagonal and late off-diagonal decoding seem similar across the different levels of decision confidence.

In Figure 3*B*, on-diagonal decoding performance is shown, plotted separately for high and low confidence decisions. We observed significantly higher decoding for the earlier perceptual response, peaking at 164–188 ms, compared to the later response (*F*_(1,22)_ = 12.95, MSE < 0.01, *p* = 0.002), in line with previous reports of sharply peaking category-selective brain responses in visual cortex, both using EEG (Bentin et al., 1996; Rossion and Caharel, 2011) and MEG (Carlson et al., 2013; Kaiser et al., 2016; Marti and Dehaene, 2017). However, interestingly, confidence was associated with classification performance only in the later, and not during the early latency window (confidence × latency interaction: *F*_(1,22)_ = 6.09, MSE < 0.01, *p* = 0.022; confidence main effect: *F*_(1,22)_ = 3.66, MSE < 0.01, *p* = 0.069). Classification accuracy for late EEG decoding was significantly better for high versus low confidence decisions (M_High_ = 0.544 vs M_Low_ = 0.513, *t*_(22)_ = 2.40, SE = 0.01, *p* = 0.025), and did not exceed chance-level for low confidence decisions (*t*_(22)_ = 1.44, *p* = 0.164). That the early brain responses were not related to decision confidence (M_High_ = 0.562 vs M_Low_ = 0.555, *t*_(22)_ = 0.80, SE = 0.01, *p* = 0.433) is consistent with predictions based on theoretical models of conscious perception (Dehaene et al., 2006; Lamme, 2006; Del Cul et al., 2007).

Crucially, off-diagonal decoding performance (trained at 150–200 ms; Fig. 3*C*) revealed a strikingly different temporal profile of results. Decoding was similarly higher early in time than later in time (*F*_(1,22)_ = 17.93, MSE < 0.01, *p* < 0.001), and significantly above chance for late off-diagonal decoding (*t*s > 2.48, *p*s < 0.021), reflecting sensory maintenance of stimulus category-specific information. However, in contrast to the on-diagonal decoding patterns, no effects of confidence were observed (confidence × latency interaction: *F*_(1,22)_ = 2.45, MSE < 0.01, *p* = 0.132; confidence main effect: *F*_(1,22)_ < 0.01, MSE < 0.01, *p* = 0.962). Thus, training on early category-selective EEG activity (150–200 ms) resulted in significant classification of later EEG activity (at 350–500 ms) that was unrelated to the level of decision confidence (M_High_ = 0.517 vs M_Low_ = 0.521, *t*_(22)_ = 0.46, SE = 0.01, *p* = 0.648).

Including all response trials (cf. only correct trials) produced an identical pattern of results at the early sensory decoding latency window. Early decoding accuracy was above chance at each confidence level (*t*s > 5.31, *p*s < 0.001), but was indistinguishable between high versus low confidence responses (*t*s > 0.71, *p*s > 0.870). This indistinguishable early decoding accuracy for high-confidence and low-confidence responses demonstrates that differences between confidence levels later in time are not attributable to differences in bottom-up (sensory) processing or SNR.

Cluster-corrected analyses of decoding accuracy against chance are shown across the entire time-window of the decoding profiles in Figure 3*B*,*C*, bottom. These analyses show that our results are not dependent on the selection of specific time-windows. In addition, decoding accuracy based on responses with the lowest indicated confidence rating (1, denoted by black crosses, right panels) revealed a very similar pattern of results to the low confidence condition (1 and 2). No statistical analyses were conducted for this lowest confidence condition as only 13 participants had sufficient trials (see Methods for details). This does however imply that one can decode category information from visual cortex early, even when participants behaviorally perform at chance (*d'* does not statistically deviate from chance for confidence level 1; Fig. 1*B*). Because mean reported confidence level toward faces was slightly different from that toward houses (M_Face_ = 2.90 vs M_House_ = 2.40, *t*_(23)_ = 5.25, SE = 0.10, *p* < 0.001), we also tested the classifier separately on confidence level 3 (*n* = 23) and confidence level 4 (*n* = 19). For both confidence levels, we observed on-diagonal and off-diagonal decoding accuracy above chance for early (*t*s > 4.46, *p*s < 0.001) and late latency windows (*t*s > 2.23, *p*s < 0.039), thereby arguing against differences in internal evidence/confidence between high/low confidence, but rather category-selective stimulus information, as driving the face versus house discrimination during the late latency window.

We then investigated whether classification performance at sensory regions (early, late on-diagonal, and late off-diagonal) may positively predict perceptual sensitivity (*d*’) and/or second-order metacognitive insight (meta-*d*’ efficiency) across participants (two-tailed Spearman correlation analyses, corrected for multiple comparisons using Holm–Bonferroni method; Holm, 1979). However, neither first-order (decoding on only correct trials: *r*s = –0.47 to –0.20, *p*s > 0.018; decoding on all trials: rs = –0.44 to –0.23, *p*s > 0.030) nor second-order measures of performance (decoding on only correct trials: *r*s = –0.23 to .26, *p*s > 0.207; decoding on all trials: rs = –0.18 to 0.21, *p*s > 0.317) were positively correlated with classification accuracy after Holm–Bonferroni correction (if anything, correlations were observed in the opposite direction than hypothesized).

Taken together, these findings support a late dissociation whereby confidence is associated with category-selective classifier performance for late on-diagonal (large square-shaped region) EEG activity, but not for late off-diagonal (rectangle-shaped region) EEG activity. Despite this differential relationship between category selective brain responses and reported confidence, such activity does not predict behavioral category discrimination performance.

### Frontal-central electrodes

We next turned to examining the relationship between confidence and category-selective decoding of later and more anterior brain responses, proposed to play an important role in metacognition (Rounis et al., 2010; Fleming and Dolan, 2012; Fleming et al., 2014). The generalization matrices in Figure 4*A* show the same drop in decoding accuracy between high and low confidence decisions in the late square-shaped region. As predicted, early (150–200 ms) frontal-central brain responses were virtually absent, and not significantly decodable (Fig. 4*A*, small square region; *t*s < 1.34, *p*s > 0.195) highlighting that perceptual/information processing is restricted to sensory areas early in time (<200 ms in this case; Del Cul et al., 2007; Marti and Dehaene, 2017).

Both on-diagonal (Fig. 4*B*) and off-diagonal (Fig. 4*C*) decoding performance are shown, plotted separately for high and low confidence decisions. Here again, late EEG decoding follows the same pattern as for the occipital-parietal electrode sets, confidence was related to classification accuracy for on-diagonal, and not off-diagonal, decoding (confidence × generalization: *F*_(1,22)_ = 6.33, MSE < 0.01, *p* = 0.020; generalization main effect: *F*_(1,22)_ = 6.17, MSE < 0.01, *p* = 0.021; confidence main effect: *F*_(1,22)_ = 2.22, MSE < 0.01, *p* = 0.150). As observed in Figure 4*B*, late classification accuracy of on-diagonal decoding was significantly better for high versus low confidence decisions (M_High_ = 0.542 vs M_Low_ = 0.512; *t*_(22)_ = 2.20, SE = 0.01, *p* = 0.039), and was not better than chance for low confidence decisions (*t*_(22)_ = 1.12, *p* = 0.275). However, late classification accuracy of off-diagonal decoding (i.e., when trained on earlier EEG activity at 150–200 ms; Fig. 4*C*) did not depend on decision confidence (M_High_ = 0.518 vs M_Low_ = 0.509; *t*_(22)_ = 0.62, SE = 0.01, *p* = 0.541). Moreover, late off-diagonal decoding for neither level of decision confidence was above chance (*t*s < 2.18). Testing the classifier separately on confidence level 3 (*n* = 23) and confidence level 4 (*n* = 19) for frontal-central electrodes, both revealed late decoding accuracy above chance for on-diagonal (*t*s > 3.53, *p*s < 0.002) but not for off-diagonal decoding (*t*s < 1.58, *p*s > 0.131).

Finally, we examined whether on-diagonal and off-diagonal decoding of late frontal-central EEG activity predicted behavioral performance. Classification performance was not correlated with first-order perceptual sensitivity (decoding on only correct trials: *r*s = 0.03–0.18, *p*s > 0.384; decoding on all trials: *r*s = –0.11 to 0.09, *p*s > 0.612). However, we observed a significant positive correlation between on-diagonal classification performance and second-order metacognitive insight (decoding on only correct trials: *r*_23_ = 0.61, *p* = 0.002, bootstrapped 95% CI [0.31, 0.82]; decoding on all trials: *r*_23_ = 0.56, *p* < 0.005, bootstrapped 95% CI [0.16, 0.77]). Participants who were more metacognitively efficient maintained stronger and more stable category-selective representations in frontal-central areas, consistent with the research showing these areas contribute to metacognitive insight (Rounis et al., 2010; Fleming and Dolan, 2012; Fleming et al., 2014). This relationship did not hold for off-diagonal activity (decoding on only correct trials: *r*_23_ = 0.38, *p* = 0.061; decoding on all trials: *r*_23_ = 0.28, *p* = 0.171).

We focused our analyses on a fronto-central region of interest (ROI) for two reasons: (1) metacognitive insight is associated with anterior parts of PFC, and (2) this creates non-overlapping ROIs for our analyses. The critical interaction between confidence and generalization type for classification performance, and the pattern of correlations with first- and second-order discrimination behavior held even when using a more posterior and data-driven electrode set (for further details on selection method, see Materials and Methods).

In summary, both occipital-parietal and frontal-central electrode sets uncovered later decodable activity that was related to confidence when a classifier was also trained on late EEG activity (on-diagonal, large square), but not when the classifier was trained on early EEG activity (off-diagonal, rectangle). This late dissociation suggests two functionally independent category-selective representations of the stimulus temporally co-exist, one that is related to confidence and one that is not. Here, the frontal-central brain responses related to reported confidence, also predicts higher levels of behavioral metacognitive performance.

### Expectation results

A secondary focus of the present research was to determine the role of expectation on category-selective processing. Specifically, we investigated whether a bi-interpretable stimulus is more likely interpreted according to the expected or unexpected stimulus category. However, expectation did not modulate the interpretation of bi-interpretable stimuli. Behaviorally, there was an overall preference to respond house (63% of responses), but this was independent of the preceding tone type (expect-face vs neutral vs expect-house cue; M = 0.61–0.67, SD = 0.10–0.11; *F*_(1.64,39.37)_ = 2.77, MSE = 0.01, *p* = 0.085). Similarly, we were unable to decode any on-diagonal neural activity from these stimuli based on preceding expect-face versus expect-house cues (*t*s < 1.36). This shows that, with the present methodology, we could not find an effect of expectation on the processing of bi-interpretable stimuli.

For single stimulus category images, we observed a significant effect of expectation on *d’* (*F*_(1.30,31.29)_ = 4.96, MSE = 0.18, *p* = 0.025), driven by a significantly lower *d’* for unexpected versus expected stimuli (*t*_(24)_ = 2.24, SE = 0.13, *p* = 0.035). These data show some behavioral evidence that expected stimuli were more easily perceived than unexpected stimuli. However, there was no significant impact of expectation on meta-*d*’ efficiency (*F*_(1.25,29.88)_ = 1.07, MSE = 0.31, *p* = 0.325). On-diagonal decodability of the single stimulus category was not modulated by expectation (unexpected vs expected) for either occipital-parietal (*F*s < 1.58) or frontal-central electrode sets (*t*_(24)_ = –1.01, *SE* < 0.01, *p* = 0.282). Finally, we found that expectation, as defined by validity of the cue (unexpected vs expected), was not decodable in itself (*t*s < 0.95).

Together, these data indicate that expectation, as rendered by the tones, had little impact on either behavioral performance or electrophysiological activity. We elaborate further on the lack of any significant findings regarding expectation in the Discussion section.

## Discussion

In a task where participants discriminated masked faces and houses and indicated their confidence in these decisions, we observed three distinct types of category-selective neural activity patterns in human EEG traces. These patterns were dissociable by their timing, scalp topography, relationship with decision confidence, and temporal generalization profile. First, we observed an early peak in classification accuracy (150–200 ms), unique for occipital-parietal electrodes and unrelated to decision confidence. This decoding profile reflects relatively early extraction of category-selective features in posterior brain regions, observed previously (Carlson et al., 2013; Kaiser et al., 2016; Marti and Dehaene, 2017). Early decoding was independent of the selection of trials used for decoding (whether including only correct trials or all trials). Later in time (∼350–500 ms), two additional independent, but co-occurring, decodable patterns of neural activity were observed. A sustained period of on*-*diagonal classification (training and testing on the same EEG signal) was observed at both occipital-parietal and frontal-central electrode sets. Intriguingly, this activity was strongly related to decision confidence and predictive of an individual’s metacognitive insight into their first-order perceptual decision (face/house discrimination). We would like to note however that small-n correlations should be interpreted with caution (Yarkoni, 2009). As such, this widely observed signal may reflect the global ignition of a broad fronto-parietal network (e.g., Global Neuronal Workspace; Dehaene and Naccache, 2001; Dehaene et al., 2006) crucial for the cognitive maintenance of category-specific stimulus characteristics, related to conscious access of the stimulus. Interestingly, later off*-*diagonal classification performance was prominent for occipital-parietal electrodes only. A classifier trained on early sensory signals could generalize later in time and this activity pattern was indistinguishable for high-confidence and low-confidence responses. Thus, this activity pattern likely reflects sensory maintenance of category-selective stimulus information.

Several influential theories of consciousness try to explain how, when and where conscious perception emerges from brain activity, and how this differs from processing unconscious information (Rees et al., 2002; Tononi and Koch, 2008; Haynes, 2009; Dehaene and Changeux, 2011; Kunde et al., 2012; van Gaal and Lamme, 2012; Boly et al., 2013). Although controversy exists, most theories postulate that early feedforward processing of information may be independent of conscious access and that feedback from higher-level to lower-level brain regions is crucial for conscious report. Feedback mechanisms allow information to be integrated and exchanged among different neural modules and may enable the maintenance of information over longer periods of time. Although speculative, the two distinct category-selective neural activity patterns observed here may relate to different types of theorized feedback processes. The late confidence-based (on-diagonal) pattern may indicate global cognitive maintenance processes supporting conscious reportability and confidence, whereas the other (off-diagonal) pattern, showing sensory maintenance of category-specific information that was unrelated to decision confidence, may indicate more local recurrent processes within visual (sensory) cortices. Although intriguing, future studies are necessary to confirm this interpretation of the present data.

Recent evidence, using similar decoding techniques on human electrophysiological data, has shown that brain processes multiple different stimuli (Marti and Dehaene, 2017) and stimulus characteristics (e.g., contrast, spatial frequency) in parallel (King et al., 2016), outside the scope of awareness. To illustrate, Marti and Dehaene (2017) observed sustained off-diagonal decoding of stimulus-evoked activity in an attentional blink paradigm. In their task, a classifier trained to discriminate several image categories (faces, places, body parts and objects) at 170-ms poststimulus onset, could significantly decode MEG activity as late as 720-ms poststimulus onset. This late phase of decoding was observed only for task-relevant target stimuli (maintained for later report) and those stimuli immediately preceding the target (likely related to broad attentional sampling). Consistent with our interpretations here, the authors attributed their findings to top-down reactivation of early sensory stages. Here, we find converging evidence for such relatively long-lasting sensory maintenance using EEG decoding techniques, but crucially, we extend their findings by showing that this type of sensory maintenance was unrelated to the level of reported confidence.

While the absence of on-diagonal when compared to off-diagonal decoding for low confidence trials (Fig. 3*B* vs *C*) may be partly explained by differences in strength of classifier training activity, SNR cannot itself explain why the difference between high versus low confidence decoding accuracy was selectively observed for on-diagonal and not off-diagonal decoding. Because both high and low confidence decoding accuracy was always based on training on the same trials, any differences observed during testing were therefore due to differences in the testing set (not the training set), and hence related to differences in confidence. Moreover, given that early decoding was indistinguishable between low and high confidence, the later on-diagonal decoding difference between low and high confidence (Fig. 3*B*) is unlikely attributable to SNR differences, but rather reflect a substantive phenomenon underlying the neural coding of confidence.

The timing (150–200 ms) and scalp topography of the early peak of decoding performance, where we showed initial category-selective processing, appears related to the N170 ERP component, specific to face processing (Bentin et al., 1996; Rossion and Jacques, 2011). Such peaks of N170-like decoding performance have been linked to activity in the occipital face area, superior temporal sulcus and/or the fusiform face area (FFA) in ventral-temporal cortex (Linkenkaer-Hansen et al., 1998; Halgren et al., 2000; Haxby et al., 2000; Itier and Taylor, 2004; Deffke et al., 2007; Rossion and Jacques, 2011). Based on indications that the late off-diagonal decodable activity reflects ongoing sensory maintenance, we speculate that the signal reported here originates from similar higher visual/ventral areas. Here, N170-like decoding performance was unrelated to decision confidence, consistent with research showing no modulation of N170-like components (or FFA activity) as a function of conscious reportability in continuous flash suppression (Suzuki and Noguchi, 2013), attentional blink (Harris et al., 2013), dichoptic fusion (Fahrenfort et al., 2012), and visuospatial neglect (Vuilleumier et al., 2001). However, others have observed reduced/absent N170-like processing for unseen faces presented during inattentional blindness (Shafto and Pitts, 2015), object substitution masking (Reiss and Hoffman, 2007) and backward/sandwich masking paradigms (Harris et al., 2011; Rodríguez et al., 2012). While these discrepant findings are not easily reconciled, one explanation could be that they depend on the degree to which feedforward processing and/or (local) recurrent processing in visual areas is disrupted by the manipulation used to affect stimulus awareness/reportability (Dehaene et al., 2006; Breitmeyer, 2015). However, the degree to which (early) face processing and category-selective visual processing, in general, is affected by different masking procedures, merits further experimentation to substantiate this suggestion.

Previous neuroimaging studies have linked the ability to estimate decision confidence to structural (Fleming et al., 2010) and functional (Fleming et al., 2012, 2014; Gherman and Philiastides, 2018) properties of the anterior prefrontal cortex. Here, we find only later category-selective decodable activity (350–500 ms) over the frontal-central electrodes predicted an observer’s metacognitive performance. Perceptual and confidence decisions were traditionally assumed to occur simultaneously, with confidence reports based on the same information relative to the same evidence continuum used for perceptual decisions (Kepecs et al., 2008; Kiani and Shadlen, 2009). Recent work has challenged this interpretation by suggesting that perceptual decisions and confidence are dissociable both neurally and behaviorally (Zylberberg et al., 2012; Maniscalco et al., 2016; Peters et al., 2017). Such work finds that while perceptual decisions are based on an optimal balance of evidence, observers are suboptimal in their metacognitive sensitivity/insight, whereby confidence in a decision is more heavily weighted by evidence for (vs against) a selected perceptual decision. The broad fronto-central distribution of the late decoding pattern (350–500 ms) that we have linked to decision confidence in the current study (for similar findings, see Gherman and Philiastides, 2015, 2018) is reminiscent of similar signals observed in previous EEG studies related to several cognitive processes associated with challenging perceptual decisions. For example, a similar broad central parietal positivity (CPP) has been shown to scale with the amount of evidence accumulated toward a decision (O’Connell et al., 2012; Kelly and O’Connell, 2013), subjective ratings of stimulus visibility during perceptual decisions (Del Cul et al., 2007; Tagliabue et al., 2018), and improvements in postsensory processing due to category-selective perceptual learning (Diaz et al., 2017). It may be that the signals that we measure on the scalp with EEG reflect a mixture of decision processes, including evidence accumulation, confidence computation, and error monitoring (Boldt and Yeung, 2015), with further studies required to disentangle and pinpoint the neural signatures of each process.

Finally, whether a particular semantic category was expected or unexpected had little bearing on how a stimulus was processed and reported in our study. The lack of both electrophysiological and behavioral evidence suggests that, generally, participants did not use the trial-wise tones in their category discrimination decisions. While cue tones could assist participants in their discrimination decisions, they were not necessary for performing the perceptual discrimination task, and so may explain the lack of expectation effects. It is however notable that others (for review, see Summerfield and De Lange, 2014) observed an impact of expectation on low-level visual processing using a near identical tone-cuing procedure. Future work related to the role of task-relevance, training, automaticity and/or motivational aspects related to task performance on the (absence of) effects of expectation on sensory processing may shed further light on this issue (see also Slagter et al., 2018).

## Synthesis

Reviewing Editor: Li Li, New York University Shanghai Decisions are customarily a result of the Reviewing Editor and the peer reviewers coming together and discussing their recommendations until a consensus is reached. When revisions are invited, a fact-based synthesis statement explaining their decision and outlining what is needed to prepare a revision will be listed below. The following reviewer(s) agreed to reveal their identity: Jason Samaha, Sabina Gherman.

The manuscript was accepted after editorial review.
